# cAMP promotes the differentiation of neural progenitor cells *in vitro* via modulation of voltage-gated calcium channels

**DOI:** 10.3389/fncel.2013.00155

**Published:** 2013-09-19

**Authors:** Guilherme Lepski, Cinthia E. Jannes, Guido Nikkhah, Josef Bischofberger

**Affiliations:** ^1^Department of Functional and Stereotactic Neurosurgery, University Albert-LudwigFreiburg, Germany; ^2^Department of Neurosurgery, Eberhard Karls UniversityTübingen, Germany; ^3^Institute of Cardiology, Universidade de São PauloSão Paulo, Brazil; ^4^Department of Stereotactic Neurosurgery, University Clinic ErlangenErlangen, Germany; ^5^Department of Biomedicine, Institute of Physiology, University of BaselBasel, Switzerland; ^6^Department of Physiology, University Albert-LudwigFreiburg, Germany

**Keywords:** neural stem cells, cell differentiation, patch-clamp techniques, calcium signaling, cyclic AMP

## Abstract

The molecular mechanisms underlying the differentiation of neural progenitor cells (NPCs) remain poorly understood. In this study we investigated the role of Ca^2+^ and cAMP (cyclic adenosine monophosphate) in the differentiation of NPCs extracted from the subventricular zone of E14.5 rat embryos. Patch clamp recordings revealed that increasing cAMP-signaling with Forskolin or IBMX (3-isobutyl-1-methylxantine) significantly facilitated neuronal functional maturation. A continuous application of IBMX to the differentiation medium substantially increased the functional expression of voltage-gated Na^+^ and K^+^ channels, as well as neuronal firing frequency. Furthermore, we observed an increase in the frequency of spontaneous synaptic currents and in the amplitude of evoked glutamatergic and GABAergic synaptic currents. The most prominent acute effect of applying IBMX was an increase in L-type Ca^2+^currents. Conversely, blocking L-type channels strongly inhibited dendritic outgrowth and synapse formation even in the presence of IBMX, indicating that voltage-gated Ca^2+^ influx plays a major role in neuronal differentiation. Finally, we found that nifedipine completely blocks IBMX-induced CREB phosphorylation (cAMP-response-element-binding protein), indicating that the activity of this important transcription factor equally depends on both enhanced cAMP and voltage-gated Ca^2+^-signaling. Taken together, these data indicate that the up-regulation of voltage-gated L-type Ca^2+^-channels and early electrical excitability are critical steps in the cAMP-dependent differentiation of SVZ-derived NPCs into functional neurons. To our knowledge, this is the first demonstration of the acute effects of cAMP on voltage-gated Ca^+2^channels in NPC-derived developing neurons.

## Introduction

Previous studies have reported the generation (*in vitro*) of functional neurons from neural stem cells/progenitor cells derived from fetal (Auerbach et al., 2000), neonatal (Scheffler et al., 2005), and adult (Song et al., 2002) brain tissue. It is well-known that neural stem cell proliferation and differentiation depend on several growth factors, hormones, and neurotransmitters. Nevertheless, the precise molecular mechanisms underlying neurodifferentiation remain poorly understood.

The activation of many G-protein-coupled receptors stimulates cAMP production as well as CREB [cAMP response element (CRE) binding protein] phosphorylation, two processes believed to be important for NPC proliferation and differentiation (Dworkin and Mantamadiotis, 2010). For adult neural stem cells, (Fujioka et al., 2004) showed that prolonged cAMP elevation induced by Forskolin increases the length and number of dendritic branches as well as the number of MAP2ab (microtubule-associated-protein 2ab) positive cells during the first 2 weeks of differentiation. Furthermore, cAMP's effects on morphological maturation are believed to depend on CREB activation (e.g., via phosphorylation by PKA at Ser-133; Jagasia et al., 2009). Once activated, CREB can bind to the promoter region of genes containing CRE. Whereas phosphorylated CREB (pCREB) is basically absent in stem cells, it is transiently up-regulated in NPCs and young neurons during the first few weeks of differentiation (Merz et al., 2011). The amount of available pCREB during this time period correlates with the total dendritic length and the morphological maturity of the young neurons (Giachino et al., 2005), while the expression of dominant negative pCREB has been shown to strongly interfere with their survival and differentiation (Herold et al., 2011).

In addition to PKA, other Ca^2+^-dependent protein kinases are known to be potent activators of CREB-mediated gene transcription, for example, mitogen-activated protein kinase (MAPK) and Ca^2+^calmodulin-dependent protein kinase IV (CaMKIV), found predominantly in the nucleus (Deisseroth et al., 2003). (Redmond et al., 2002) showed that CREB phosphorylation is mediated by CaMKIV and blocked by L-type Ca^2+^ channel antagonists. Consistent with these observations, NPC-derived neurons express L-type Ca^2+^ channels a few days after mitosis and show spontaneous Ca^2+^ oscillations, which have been shown to support neuronal differentiation (D'Aascenzo et al., 2006). Much less is known about the impact of Ca^2+^ channel modulation on the functional properties of NPCs. Most importantly, almost nothing is known about the functional interaction between cAMP- and Ca^2+^-dependent pathways during the differentiation of NPCs into functional neurons.

In the current study we investigated the crosstalk between cAMP and Ca^2+^-signaling during the differentiation of SVZ-derived NPCs. We independently tested the effects of BDNF, NT3, IBMX, and Forskolin on neuronal differentiation. Our results revealed that applying the phosphodiesterase inhibitor IBMX elevates cAMP levels, which in turn strongly accelerates the generation of action potentials and the development of functional inhibitory and excitatory synapses. Enhanced cAMP levels increased Ca^2+^ influx via the voltage-gated L-type Ca^2+^ channels, which in turn facilitated CREB phosphorylation. This proved to be a critical step toward neuronal differentiation and the formation of functional synapses.

## Materials and methods

### Cell culture

**Figure A1:**
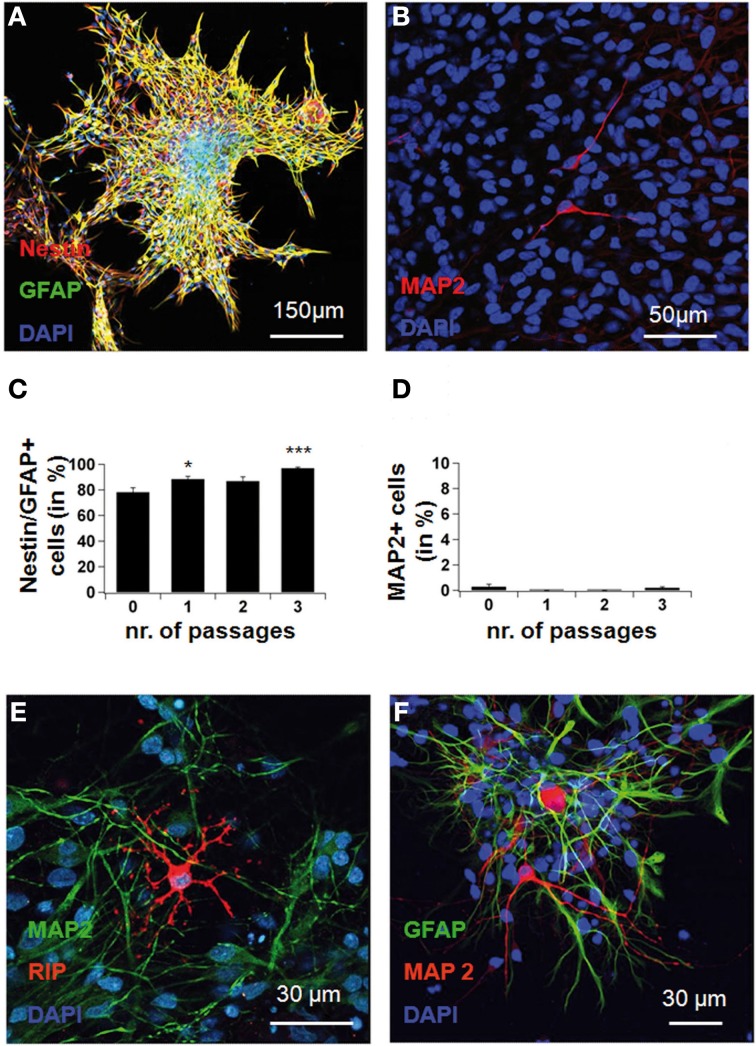
**Neural stem cells isolated before differentiation**. Cells were expanded *in vitro* until the formation of free-floating neurospheres. These were plated onto poly-L-lysine, and 2 h after plating, were fixed and immunostained for nestin, GFAP and MAP2 after 0, 1, 2, 3, and more passages. In **(A)**, confocal picture showing nestin and GFAP co-expression in a culture expanded for 3 passages. Note that cells adhere to the culture flask and migrate away from the core of the spheres. In **(B)**, MAP2ab-staining in the same culture showing that the occurrence of MAP2-positive neurons before differentiation is extremely rare. In **(C)**, the yield of progenitor cells, double-stained for nestin and GFAP, rose gradually with time in expansion culture, as mature cell types died. In **(D)**, no significant MAP2-positivity was seen in the system before differentiation. In **(E)**, confocal microscopy after differentiation in medium rich in cAMP, showing oligodendrocytes (anti-RIP, revealed in red) and neurons (anti-MAP2, revealed in green). In **(F)**, astrocytes (anti-GFAP, green) and neurons (anti-MAP2ab, red). Red represents AlexaFluor568 and green represents AlexaFluor488.

All experiments were approved by the Research Ethics Committee of the Albert-Ludwig University, in Freiburg. Neural stem cells were originally isolated from E14.5 Sprague-Dawley rat fetuses, as previously described (Lepski et al., 2011a). Pregnant females were anesthetized with an intraperitoneal injection of ketamine (Essex Pharma, Munique), the fetuses were extracted, the telencephalic vesicle was then dissected and digested with trypsin 0.05% (Worthington, Lakewood, New Jersey, USA) and DNAse 0.05% (Sigma, St. Louis, Missouri, USA). After mechanical trituration, the resulting suspension was cultured in medium comprising DMEM, F12, 2 mM L-Glutamine, 2% B27 serum supplement, 1% PSA (all purchased from Gibco), 20 ng/mL basic fibroblast growth factor (bFGF, Sigma), 20 ng/mL epidermal growth factor (EGF, PeproTech, London, UK), 5 μg/mL heparin (Sigma), and incubated at 37°C, 5% CO_2_, 21% O_2_, and 95% humidity. Medium was changed every other day, and passages performed once a week by light mechanical dissociation of the formed spheres. Cultures of neural stem cells between passages 3 and 5 were used in this study. For neuronal differentiation, a previously tested protocol for human fetal neuronal progenitors was employed (Lepski et al., 2010). NPCs isolated according to this protocol were able to generate mature neurons, oligodendrocytes and astrocytes in differentiation medium (Figure A1). After a minimum of 3 passages, cells were plated at high densities on poly-L-ornithine (Sigma) coated coverslips placed into 24-well plates in a medium comprising MEM (Sigma), 1% N2 serum supplement, 1% sodium pyruvate, 2 mM L-glutamine, 4 mM glucose, 0.1% bovine serum albumin, 1% PSA, supplemented with BDNF 25 ng/mL and 0.5 mM3-isobutyl-1-methylxantine (IBMX, Sigma). In some experiments, 10 ng/mL Neurotrophin-3 (NT3, Sigma) and 10 μM Forskolin (Sigma) were added separately to the differentiation medium, as described in the results. In order to block neuronal differentiation, 50 μM Ni and 100 nM TTX, 200 μM EGTA (Sigma), 50 μM Cd (Merck, Darmstadt, Germany), 10 μM nifedipine (Sigma) were added to the medium containing IBMX throughout the differentiation period. Medium was changed completely every other day. Cells were fixed and immunostained immediately after electrophysiological recording, at different time points during the first week or on the seventh day.

### Electrophysiology

At different time points during the differentiation process (at 1,3,5, and 7 days *in vitro*), the cultured coverslips were transferred to a recording chamber and continuously superfused with physiological extracellular saline at a flow rate of 5–10 mL/min (chamber volume ~2 mL). Neuronal cells were visually identified based on the presence of short or long neuritic elongations. Cells with GAP junctions, which are present in astrocytes, were discarded if patched accidentally. Further details related to the electrophysiological methods have been published previously (Lepski et al., 2010). The saline contained (in mM): 125 NaCl, 25 NaHCO_3_, 2.5 KCl, 1.25 NaH_2_PO_4_, 25 Glucose, 2 CaCl_2_, and 1 MgCl_2_ (equilibrated with 95% O_2_, 5% CO_2_). An Axopatch 200 A amplifier (Axon Instruments, Foster City, CA) was used for voltage-clamp and current-clamp (I-clamp fast) recordings. Current and voltage signals were filtered at 5–10 kHz, with a 4-pole low pass Bessel filter and digitalized at 10 or 20 kHz with a 1401 plus interface (CED, Cambridge Electronic Design, Cambridge, UK) connected to a personal computer. Only cells with seal resistance (R_seal_) larger than three times the input resistance (R_in_) were used in this study.

For whole cell current clamp recordings, we used 4–9 MΩ patch pipettes filled with an internal solution containing (in mM): 120 K-gluconate, 20 KCl, 10 EGTA, 2 MgCl_2_, 2 NA_2_ATP, 10 HEPES (pH adjusted to 7.3 with KOH). Bridge balance was used to compensate the series resistance of 20–40 MΩ. In addition, capacitance compensation was used to decrease the charging time of the pipette to less than 50 microseconds. The holding potential was set to −80 mV by applying a small hyperpolarizing holding current if necessary.

Currents were evoked by applying 15 voltage pulses from a holding potential of −80 mV, starting at −70 mV with 10 mV increments up to +70 mV. Leak and capacitive currents were subtracted using a P/-4 protocol, as described elsewhere (Lepski et al., 2010). All experiments were performed at 22°C. Na^+^- and K^+^-peak currents were calculated from 5 and 100 data points, from the minimal and maximal curve values, respectively. Cell capacitance was calculated by mono-exponential fitting from the averaged current trace, by applying 50 pulses of −10 mV and 100 ms from a holding potential of −80 mV.

Ca^2+^-current measurements were taken by filling the pipette with an internal solution containing (in mM): 137 CsCl, 4 MgCl_2_, 10 EGTA, 10 HEPES, 4 Na_2_ATP, 10 Na_2_-phosphocreatin, 0.3 NaGTP (pH adjusted to 7.3 with CsOH). The bath solution used in these experiments contained (in mM): 105 NaCl, 25 NaHCO_3_, 25 glucose, 2.5 KCl, 1.25 NaH_2_PO4, 2 CaCl_2_, 1 MgCl_2_, 1 μM tetrodotoxin, 20 tetraethylammonium chloride, and 4 4-aminopyridine to block voltage-gated Na^+^ and K^+^ channels, respectively.

To measure Ca^2+^-currents, we used Cd^2+^ (50 μM) to nonspecifically block all Ca^2+^ channels, Ni^2+^ to block T-type (50 μM), nifedipine (10 μM) was used to block L-type high-voltage activated channels, ω-conotoxin GVIA (1 μM) to block N-type, SNX482 (100 nM) to block R-type, and ω-agatoxin IVA (250 nM) to block P/Q-type Ca^2+^ channels. All chemicals were purchased from Alomone Labs (Jerusalem, Israel).

Recordings of evoked synaptic currents were performed with a bipolar macroelectrode using voltage pulses with a duration of 200 microseconds and an amplitude of 1–10 V (5 stimuli at 10 Hz). For pharmacological identification of the evoked GABAergic and glutamatergic synaptic currents, 10 μM bicuculline methiodide and 10 μM (CNQX) were added to the external saline to block GABA_A_ and AMPA receptors, respectively.

During the recording session, cells were filled with biocytin (Molecular Probes, 1 mg/mL). All patched cells were fixed and double-stained for biocytin and a neuronal marker, MAP2 or β III-tubulin, for phenotypic confirmation.

For data analysis, traces and data points were fitted using a nonlinear least-squares algorithm. Sodium and calcium peak currents were determined based on 5 data points at the maximum inward current value. Similarly, the potassium peak current was calculated from 100 data points at the maximal outward current value. Current densities were calculated by dividing the peak current by the cell capacitance. The evoked synaptic currents were averaged and subtracted from the currents recorded during pharmacological blocking (bicuculline and CNQX) in order to reduce the stimulation artifact. To analyze the decay phase, synaptic currents were fitted with a bi-exponential function and the amplitude-weighted time constant τ_w_ was calculated according to the following equation:

(1) $$\tau_{\text{w}}=\left(A_{1}\tau_{1}+A_{2}\tau_{2}\right)\text{​}/\text{​}\left(A_{1}+A_{2}\right)$$

where *A*_1_ and τ_1_ represent amplitude and time constant from component 1, and *A*_2_ and τ_2_ for component 2.

The steady state activation curves of voltage-gated channels were obtained from the respective peak currents, assuming ohmic behavior. The activation of Na^+^- and K^+^-currents (I–V relationship) was fitted with the following equation derived from the Boltzmann equation multiplied with a driving force, considering the reversal potential for K^+^ and for Na^+^, calculated as −95 mV and 80 mV, respectively, according to the Nernst equation with the solutions used.

(2) $$I(V)=\left[\left(V-E\right)G_{\text{max}}\right]\text{​}/\text{​}\left\{1+\text{ exp}\left[-\left(V-V_{1/2}\right)\text{​}/k\right]\right\}$$

where *V* is the membrane potential, *E* is the Nernst reversal potential, *G*_max_ is the maximal conductance, *V*_1/2_ is the potential at which the value of the Boltzmann function is 0.5, and *k* is the slope factor.

The I–V relationships for Ca^2+^ currents were fitted with a modified Goldman-Hodgkin-Katz equation of the form:

(3) $$I{\left(V\right)}_{\text{Ca}}=PV\left(\left[D-\text{exp}\left(-V/C\right)\right]\text{​}/\text{​}\left[1-\text{exp}(V/C)\right]\right)*\;\left(1/\{1+\text{ exp}\left[\left(V_{1/2}-V\right)\text{​}/k\right]\}\right)$$

with voltage *V*, amplitude factor *P*, steepness factor *C*, and parameter *D* determining current rectification and reversal potential.

### Immunocytochemistry and confocal imaging

Immunostaining of cell cultures was carried out as previously described (Lepski et al., 2010, 2011b). The following primary antibodies and dilutions were used (ms, mouse; rb, rabbit): anti-MAP2ab (1:200, ms, Chemicon), anti-MAP2 (1:600, rb, Chemicon), anti-synaptophysin (1:1000, ms, Chemicon), anti-GFAP (1:600, rb, DAKO), anti-nestin (1:200, ms, Chemicon), anti-RIP (1:1000, ms, Chemicon), anti-pCREB (1:1000, mouse monoclonal, Chemicon). The secondary antibodies used were Alexa 488 goat anti-rabbit or anti-mouse (1:150, Molecular Probes), Alexa 594 goat anti-rabbit or anti-mouse (1:150, Molecular Probes); biocytin staining was revealed by fluorescein-avidin D (1:500, Vector Laboratories, Burlingame, CA). Nuclei were stained with 4,6-diamidino-2-phenylindole dihydrochloride (DAPI, 1:10,000, Sigma). Photographs were taken on a Leica TCS SP2 confocal system (405 nm diode, Ar/ArKr, HeNe 543/594 lasers), with 20x/0.7 (air) or 63x/1.2 (oil) objectives. One digital frame comprised an area of 0.562 mm^2^. For quantifications, ten consecutive images were systematically scanned from left to the right at the horizontal diameter of each coverslip for each culture condition corresponding to about 5% of the entire surface area of the coverslips (113 mm^2^). Cells positive for a specific marker were counted in highly magnified digital frames, scanned at 4 μm to avoid overprint, in relation to the total amount of DAPI-stained nuclei, using ViewFinder 2.1 software (ERDAS, Atlanta). For quantification of fluorescence intensity, experiments were performed in parallel and the groups to be compared were stained in one session. Measurements of fluorescence levels were performed on the same day using a Leica TCS SP2 confocal system. The laser parameters were kept constant during all measurements.

In addition to the number of immunopositive cells, we quantified the relative immunofluorescence for MAP2ab and synaptophysin. To do so, we measured the background fluorescence intensity F0 from a cell-free region of interest (ROI) within each individual image. Subsequently, the relative mean fluorescence intensity (F − F0)/F0 of the total image was calculated using the Leica SP2 software. Experiments were done in triplicate and counts were performed in a double-blind manner.

### Cell viability assay

To assess cell viability under different culture conditions, we used the Live/Dead Cell Viability Kit (Molecular Probes). The test is based on differential membrane permeability of the two kit components, Syto-10© green fluorescent nucleic acid stain, which is highly permeable to all cells, and Dead Red©, an ethidium homodimer-2, which is a cell impermeant dye that labels only cells with compromised membranes. Initially, a working solution containing 2 uL of each component diluted in 1 mL HBSS (HEPES-buffered saline solution) is prepared. The solution is comprised of 135 mM NaCl, 5 mM KCl, 1 mM MgSO_4_, 1.8 mM CaCl_2_, 10 mM HEPES, pH 7.4. Thereafter, 10^6^ cells in culture medium are transferred to Falcon tubes and centrifuged at 250 g for 10 min. The medium is completely removed and cells are re-suspended in 200 uL of the diluted dye. Cells are then incubated in darkness at room temperature for 15 min. Next, cells are pelleted again by centrifugation, the supernatant is carefully removed, and cells are re-suspended in a minimum volume of 50 uL fresh HBSS. This is followed by fixation for over 1 h in 4% fresh prepared glutaraldehyde. Finally, the fixative is removed and cells are re-suspended in HBSS prior to observation and quantification.

### Statistical analysis

Electrophysiological data were analyzed and fitted using Igor 5.03 (Wavemetrics). All data are expressed as mean ± s.e.m. where error bars in the figures also represent s.e.m.; the statistical significance was assessed with a two-tailed Mann–Whitney U test at the significance level (*p*) indicated (^*^p < 0.05, ^**^p < 0.01, and ^***^p < 0.001).

## Results

### cAMP-induced neuronal differentiation

**Figure 1:**
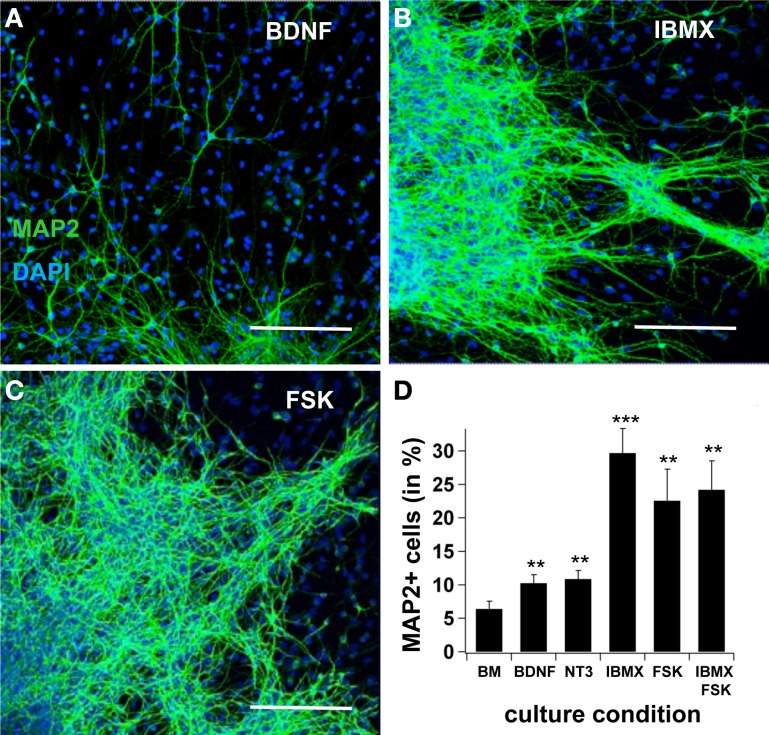
**cAMP induces neuronal differentiation**. In **(A)**, **(B)** and **(C)**, confocal pictures showing MAP2ab-positivity in cultures maintained in BDNF, IBMX, and Forskolin after 7 days, with pictures taken at the border of the spheres. In **(D)**, quantification of MAP2+-cells after 7 days in basal medium, BDNF alone, IBMX, Forskolin and IBMX and Forskolin. The highest yield of neuronal cells was observed in IBMX, and adding Forskolin did not increase the yield of differentiated cells. Green is AlexaFluor488. Scale bar 150 μm.

First, we tested neural differentiation under the effects of BDNF, NT3, IBMX, or Forskolin. In basal medium, the yield of MAP2ab-positive cells after 7 days increased from 6.4 ± 1.2% to 10.3 ± 1.3 when BDNF was added to the medium (*n* = 10, *p* < 0.05). Likewise, adding NT3 increased cell production to 10.9 ± 1.3% (*n* = 10, *p* < 0.01). A significantly greater increase in the number of MAP2ab positive cells was obtained when the intracellular cAMP concentration was elevated by adding the adenylatecyclase-activator Forskolin (22.6 ± 4.7%; *n* = 10, *p* < 0.001) or the phosphodiesterase inhibitor 3-isobutyl-1-methylxanthine (IBMX) (29.7 ± 3.7%; *n* = 10, *p* < 0.001). Interestingly, no summation effect was observed when Forskolin and IBMX were simultaneously added to the system (24.2 ± 4.3%, *n* = 10, *p* < 0.01), suggesting a saturation effect (Figure 1).

**Figure 2:**
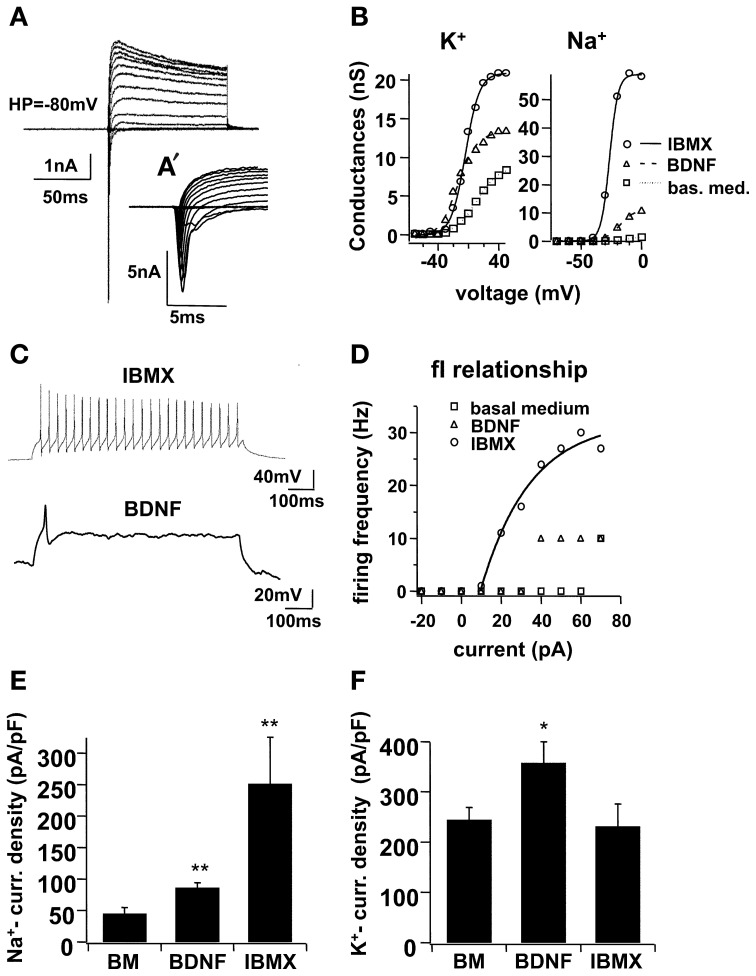
**Electrophysiological maturation of NPCs in high cAMP (IBMX), with voltage-dependent ionic currents and action potentials**. In **(A)**, voltage clamp recordings on NSCs differentiated for 7 days in IBMX. Holding potential −80 mV, 100 ms pulse duration from −70 to 70 mV at 10 mV increments, with an average of 5 consecutive sweeps. Leak and capacitive currents were subtracted using a P/-4 protocol (see text). In **(A′)**, the same Na^+^-currents from **(A)** are shown in an expanded time frame. In **(B)**, voltage-dependent activation of K^+^- and Na^+^-conductance for representative cells cultivated in basal medium, BDNF and IBMX for 7 days, fitted with a Boltzmann equation of the form *f(V)* = 1/(1 + exp[(*V*_1/2_ − *V*)/*V*_slope_]). In **(C)**, trends of action potentials induced in current clamp of a cell cultivated in IBMX, and for comparison, one simple potential evoked in a cell cultivated with BDNF; pulse duration 1 s, −20 to 70 pA at 10 pA increments, both figures represent a simple trace at +40 pA. In **(D)**, frequency-current (fI) relationship for the sample cell and for two other representative cells cultivated in basal medium and in BDNF alone. In **(E)** and **(F)**, Na^+^- and K^+^-current densities, respectively, were calculated across three different culture conditions, basal medium, BDNF and IBMX; for this, cell capacitance was calculated from a mono-exponential curve fitting in voltage-clamp mode, using a −10 mV hyperpolarizing pulse, repeated in 50 sweeps and averaged. Na^+^- and K^+^- peak currents were determined from 5 and 100 data points, respectively, from the I–V traces (5 sweeps).

Immunocytochemical markers revealed the presence of morphologically mature neuronal cells at the end of the 1-week differentiation period, but the functional properties of these cells were not yet evident. In a previous study, our group demonstrated that functional maturation may be delayed in relation to morphological development (Lepski et al., 2011b); we therefore performed electrophysiological patch-clamp recordings on the seventh day of differentiation. In voltage clamp mode, larger Na^+^- and K^+^-currents were recorded in IBMX (Figure 2). In basal medium, the Na^+^-peak conductance was 3.24 ± 0.57, *ns*, whereas the maximal K^+^ conductance was 14.23 ± 1.27, *ns*. When BDNF was added to the differentiation medium, conductance levels increased to 9.68 ± 1.38, *ns* (*n* = 13, *p* < 0.001) and 23.69 ± 5.18, *ns* (*n* = 13, *p* = 0.1713), respectively. Under IBMX, the peak Na^+^ and K^+^-conductance increased to 35.13 ± 7.04, *ns* (*n* = 13, *p* < 0.001) and 33.43 ± 7.41, *ns* (*n* = 13, *p* < 0.05), respectively. In current clamp mode, action potentials were evoked under IBMX in 63.2% of the cells (Figure 2). By contrast, only 21.7% of the cells cultivated in BDNF and 13.5% of the cells grown in basal medium fired action potentials.

**Figure 3:**
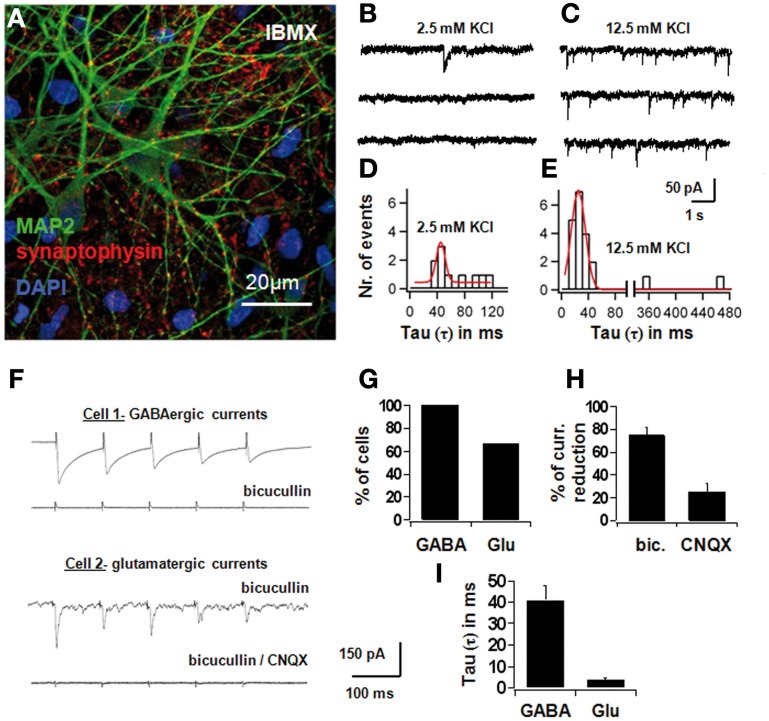
**Electrophysiological differentiation of NPCs in IBMX, with spontaneous and evoked synaptic currents**. In **(A)**, confocal picture showing MAP2 and synaptophysin positivity after 7 days in differentiation medium with IBMX. Red is AlexaFluor568 and green is AlexaFluor488. In **(B)** and **(C)**, spontaneous synaptic recordings in voltage clamp in normal saline and with increased KCl (12.5 mM). In **(D)** and **(E)**, histograms of the time constants of all synaptic events in normal saline and in increased KCl, fitted with a Gaussian curve; best-fit parameters were 37 ± 9 ms for low KCl and 20 ± 14 ms for high KCl. In **(F)**, synaptic currents in differentiated cells *in vitro* evoked through external bipolar macroelectrodes; each trace represents an average of ten consecutive signals subtracted from the stimulation artifact; stimulation parameters: 5 stimuli at 400 ms 1 to 10 V and 1 ms pulse-duration, 10 sweeps. In Cell 1, synaptic currents with long decay time were induced and blocked completely with 10 μM bicuculline methiodide, a selective GABA_A_-receptor antagonist; in cell 2, residual currents with short decay time persisted after blocking bicuculline, and this component disappeared after 10 μM 6-cyano-7-nitroquinoxaline-2,3-dione (CNQX), an AMPA-antagonist. In **(G)**, the percentage of cells with GABAergic and glutamatergic synapses, in **(H)**, the percentage of current-reduction with bicuculline and CNQX, and in **(I)** the average time constants of the bicuculline- and CNQX-sensitive synaptic components, are depicted.

On the 7th day of differentiation in the IBMX-containing medium, a larger proportion of cells expressed the mature neuronal cell marker MAP2ab and the pre-synaptic vesicle marker synaptophysin (see Figure 3A). Spontaneous synaptic activity was recorded in normal saline and with increased KCl (12.5 mM; Figures 3B–E). Synaptic current responses were evoked using a bipolar stimulation electrode, and current-components were identified with specific pharmacological blockers. A GABAergic component sensitive to bicuculline was identified in all cells, and an AMPA-component sensitive to 6-cyano-7-nitroquinoxaline-2,3-dione (CNQX) was present in 67% of the cells. Bicuculline blocked 74.6 ± 7.8% of the synaptic current, whereas CNQX blocked 25.4 ± 7.8% of the total current (Figures 3F–I).

Taken together, results showed that the functional maturation of NPCs was substantially improved by cAMP signaling. Furthermore, many more cells were able to fire action potentials and form functional glutamatergic and GABAergic synaptic contacts when IBMX was applied.

### Neuronal differentiation was impaired by tetrodotoxin and specific Ca^2+^-channel blockers

**Figure 4:**
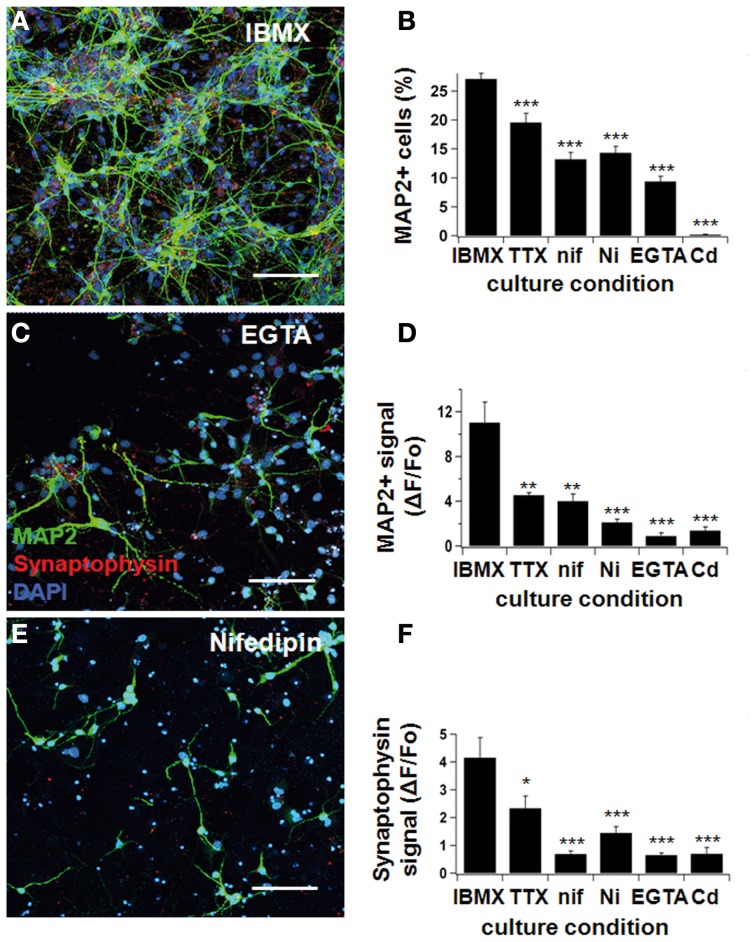
**Neurodifferentiation was impaired by Ca^2+^-blockers**. NPCs were cultivated in IBMX, as previously described. One hundred nanomolar tetrodotoxin, 10 μM nifedipine, 50 μM Ni^2+^, 500 μM EGTA, and 50 μM Cd^2+^ were added to the culture medium. After 7 days, cultures were fixed and stained for MAP2ab and synaptophysin, and pictures were taken under a confocal microscope **(A,C,E)**. Neuronal differentiation was ascertained three different ways: by counting the number of MAP2-positive cells **(B)**, by determining the fluorescence signal intensity for MAP2 **(D)**, and synaptophysin **(F)** by confocal microscopy (see text). The yield of neuronal cells was significantly reduced when Na^+^- and Ca^2+^-blockers were added to the culture medium. Green represents AlexaFluor 488 and red, AlexaFluor 568. Scale bars 150 μm.

Next, we tested neuronal differentiation in the presence of three different channel blockers: tetrodotoxin (TTX; to block Nav-channels), Cd^2+^ and Ni^2+^ (to block Cav-channels), and nifedipine (to specifically block L-type Ca^2+^channels). In addition, we applied the Ca^2+^ buffer EGTA to study the impact of intracellular Ca^2+^ signals (Figure 4).

In the presence of IBMX/BDNF, the relative number of MAP2ab positive cells was significantly decreased from 27.1 ± 1.0% to 19.6 ± 1.6% when adding TTX (*p* < 0.001, Figures 4A,B), indicating that electrical activity contributes to early NPC differentiation. An even larger effect was seen when we directly interfered with Ca^2+^ signaling, resulting in a proportion of MAP2 positive neurons of 13.2 ± 1.2% in nifedipine (*p* < 0.001), 14.3 ± 1.1% in Ni^2+^ (*p* < 0.001), 9.4 ± 0.9% in EGTA (*p* < 0.001), and 0.2 ± 0.1% in Cd^2+^ (*p* < 0.001) (see Figure 4B). The average MAP2ab- and synaptophysin-fluorescence intensity showed a similar decrease (Figures 4D,F). Therefore, blocking electrical activity or voltage-dependent Ca^2+^-influx in different ways impaired the acquisition of a mature neuronal phenotype. Most importantly, specifically blocking L-type Ca^2+^-channels with nifedipine significantly reduced differentiation and synapse formation, highlighting the importance of this type of channel in proper neuronal maturation.

### High cAMP levels increased L-type Ca^2+^-currents in developing neurons

**Figure 5:**
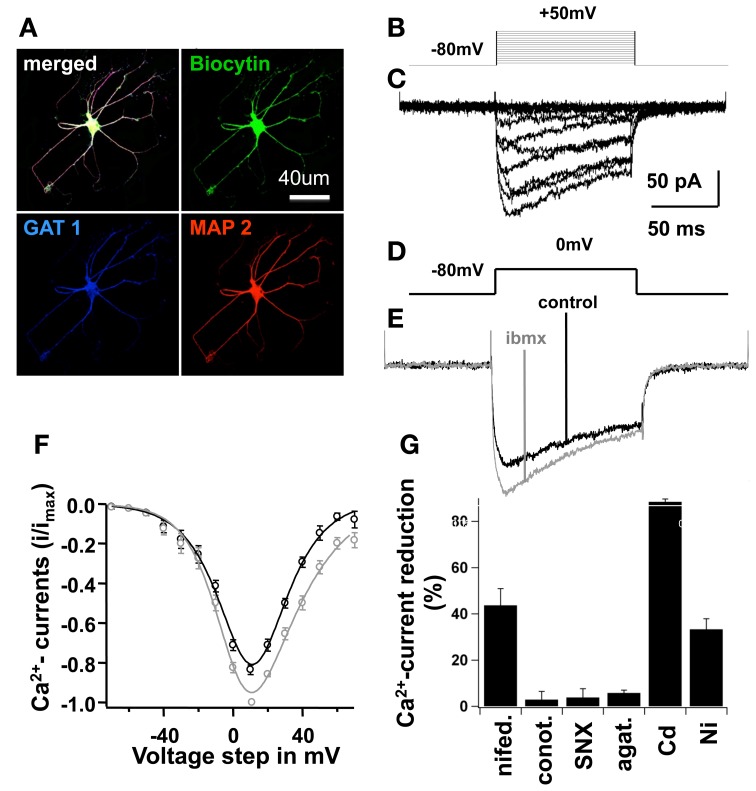
**High cAMP increased L-type Ca^2+^-currents in mature cells**. In **(A)**, an example of a mature cell is shown; this cell was filled with Biocytin during recording and further stained for MAP2 (a mature neuronal marker), and Gat-1 (GABA amino-transporter) after fixation. In (**B**), stimulation protocol in voltage-clamp, pulse duration 100 ms, holding potential −80 mV, stimuli from −70 to 50 mV at 10 mV increments. In **(C)**, voltage-clamp traces, average of 5 consecutive sweeps with subtraction of leak and capacitive currents (see text), showing Ca^2+^-influx currents. In **(D)**, voltage pulse of 0 mV to obtain traces shown in **(E)**. In **(E)**, Ca^2+^-influx was recorded in a control situation using the standard bath saline solution, and with 0.5 mM IBMX alone. Traces represent average of 10 consecutive sweeps, for at least 5 min; series resistance remained constant throughout the experiment. In **(F)**, current-voltage (I–V) relationship of Ca^2+^-influx under each condition mentioned above, bars represent SEM, data were fitted with a modified Goldman-Hodgkin-Katz equation of the form *I*(*V*) = *PV* ([*D* − exp(−*V*/*C*)]/[1 − exp(*V/C*)])*[1/(1 + exp[(*V*_1/2_ − *V*)/*V*_slope_])], see text. In **(G)**, Ca^2+^-current peaks at 0 mV were recorded with different specific Ca^2+^-blockers of L, T, P/Q and N-type Ca^2+^-channels to dissect the current-subtypes present in NSCs at this maturation phase. Bars represent percentage of current reduction in the control situation and after blocker wash-in.

In order to study the different subtypes of Ca^2+^currents found in developing NPC-derived neurons, we dissected Ca^2+^currents with specific pharmacological blockers and performed voltage clamp recordings (Figure 5). On the 7th differentiation day, L-type Ca^2+^-currents sensitive to nifedipine accounted for 43.7 ± 7.3% of the total Ca^2+^influx at a membrane potential of 10 mV.Ω-conotoxin GVIA (N-type Ca^2+^-blocker) blocked 2.9 ± 3.6% of the Ca^2+^-currents; SNX-482 (R-type blocker) blocked 3.8 ± 3.8%; ω-agatoxin IVA (P/Q-type blocker) blocked 5.8 ± 1.3%; Ni^2+^ (T-type blocker) blocked 33.3 ± 4.6%; and Cd^2+^ blocked 88.4 ± 1.3% of the total Ca^2+^current (Figure 5F). These results clearly demonstrate the predominance of L-type Ca^2+^channels in differentiated NPCs.

As shown above, the number of mature neurons derived from NPCs was significantly higher in culture conditions with increased intracellular cAMP-concentrations (IBMX or Forskolin). We also demonstrated that blocking Ca^2+^inhibits neurogenesis *in vitro*. As L-type Ca^2+^channels can be phosphorylated by PKA, we studied the effects of IBMX on whole-cell Ca^2+^currents in developing neurons. Rectangular voltage pulses evoked large Ca^2+^ currents 1 week after differentiation in IBMX and BDNF, with a maximal current amplitude of 10 mV (Figure 5). Recordings performed with and without IBMX in the bath solution showed an increased Ca^2+^current amplitude of 182 ± 31 pA in the presence of IBMX as compared to control medium (−152 ± 27 pA, *p* < 0.01, *n* = 6).

To our knowledge, this is the first demonstration of the acute effect of cAMP on voltage-gated Ca^+2^channels in NPC-derived developing neurons.

### Nifedipine blocked L-type Ca^2+^currents in differentiating neurons

**Figure 6:**
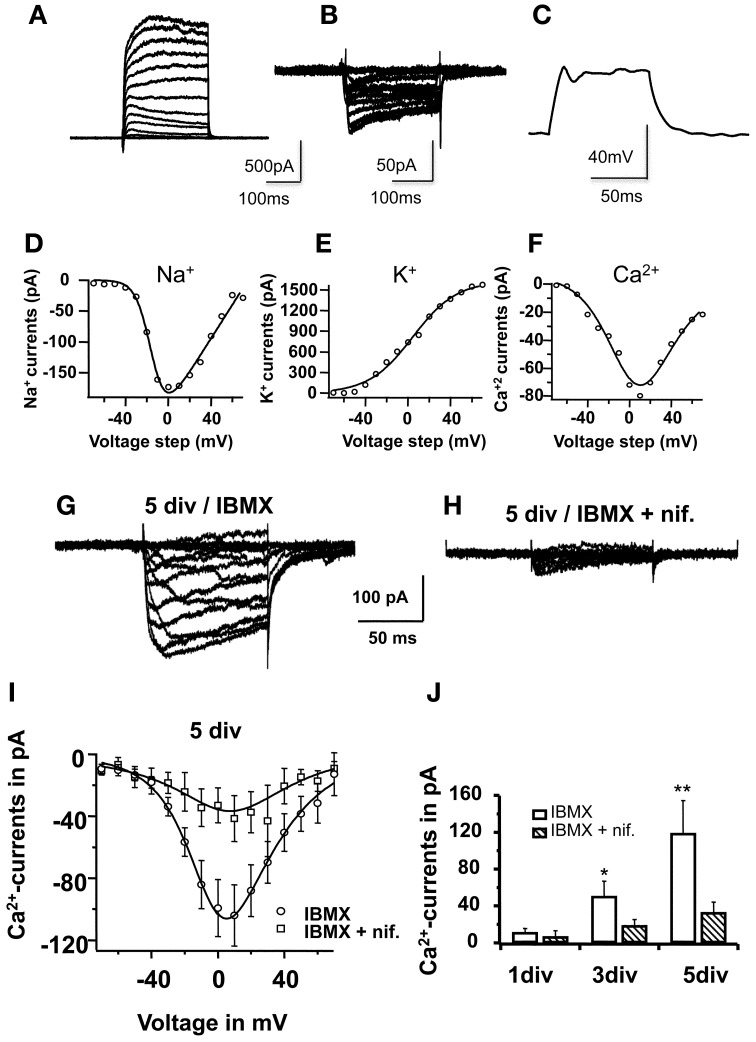
**Nifedipine blocked L-type-Ca^2+^-currents in maturing cells**. In **(A)**, voltage-clamp traces showing the I–V relationship (5 sweeps) for Na^+^ and K^+^-currents in a cell on the 2nd day *in vitro*. Note the small Na^+^ component in relation to the relatively large K^+^ component, mainly comprised of slow-rectifying K^+^-channels. In **(B)**, I–V voltage clamp traces showing Ca^2+^- influx currents in another cell also on the 2nd day *in vitro*. In **(C)**, current clamp traces after +40 pA stimulus, recorded form the same cell shown in **(A)**, showing a small voltage oscillation not sufficient to elicit an action potential. In **(E), (F)**, and **(G)**, Na^+^, K^+^, and Ca^2+^- activation curves, respectively; **(D,E)** were derived from the traces shown in **(A)**, and **(F)** was derived from the traces shown in **(B)**. All cells were recorded on the 2nd day *in vitro*. The mathematical modeling was based on the Boltzmann equation multiplied with a driving force, considering the reversal potential for K^+^ and for Na^+^, calculated as −95 mV and 80 mV, respectively, while taking the solutions used into consideration (see text). For Ca^2+^, the I–V relationships were fitted with a modified Goldman-Hodgkin-Katz equation (see text). In **(G)** and (**H**), voltage-clamp traces obtained for Ca^2+^, but on the 5th differentiation day, before complete functional maturation was achieved. In **(G)**, example of a typical recording from a cell cultivated in 0.5 mM IBMX. In **(H)**, example of a typical recording from a parallel culture in IBMX and 10 μM nifedipine. Traces represent average of 5 consecutive sweeps. In **(I)**, I–V relationship of Ca^2+^-currents under both conditions in 10 different cells; data were fitted with a modified Goldman-Hodgkin-Katz equation. In **(J)**, Ca^2+^-peak currents at 0 mV on 1st, 3rd, and 5th differentiation days, in cells cultivated with and without nifedipine. Each bar represents recordings from 10 cells.

The next question was whether the inhibitory effect of nifedipine was due to the blocking of L-type Ca^2+^channels during neuronal differentiation. To address this question, Ca^2+^currents were recorded during *in vitro* differentiation, more specifically on the 1st, 3rd, and 5th days, with and without nifedipine in the differentiation medium. Figure 6 shows typical Ca^2+^currents recorded from a cell cultivated under standard conditions (A) and in nifedipine (B). The I–V relationship of 10 pooled cells clearly demonstrated that nifedipine chronically blocked Ca^2+^influx throughout the differentiation period. An analysis of the progression of peak Ca^2+^ current at 0 mV during neuronal maturation under standard differentiation conditions revealed an increase in current: 11 ± 4 pA on the first day, 50 ± 16 pA on the third day (*p* < 0.05), and 119 ± 36 pA on the fifth day (*p* < 0.01). By contrast, when cells were constantly cultivated with nifedipine, the peak Ca^2+^ current did not increase significantly (7 ± 6 pA on the first day, 19 ± 7 pA on the third, *p* > 0.1, and 33 ± 11 pA on the fifth day, *p* > 0.1, Figure 6D). These data clearly show that in addition to blocking L-type Ca^2+^ currents, the acute application of nifedipine also inhibits the developmental up-regulation of this influx.

### Blocking of L-type Ca^2+^currents prevents CREB phosphorylation in differentiating NSCs

**Figure 7:**
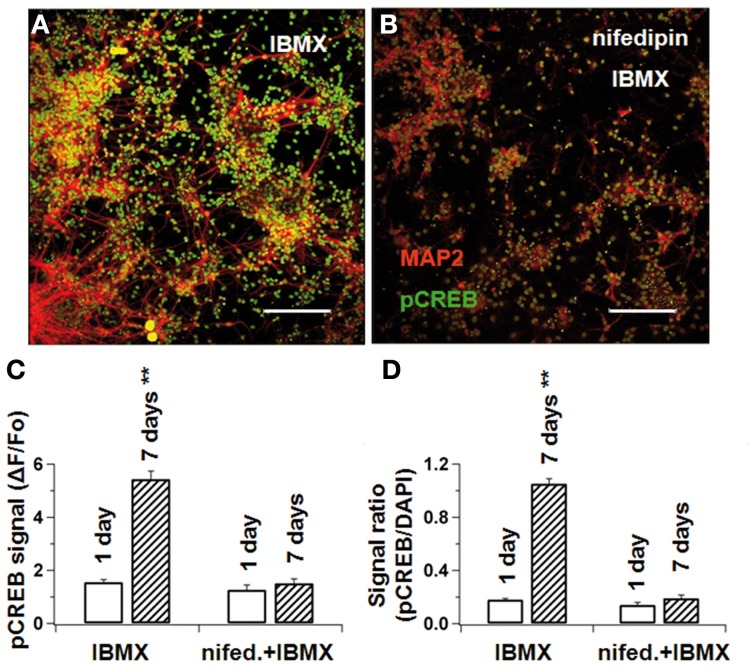
**L-type Ca^2+^-blockade prevents CREB phosphorylation in differentiating NPCs**. NPCs were cultivated either under IBMX **(A)** or IBMX with nifedipine **(B)**. After 1 and 7 days, cultures were fixed and stained concomitantly for MAP2ab and phosphorylated CREB. pCREB-signal intensity was measured by confocal microscopy. In **(C)**, pCREB signal at the beginning and end of differentiation in cultures with IBMX and IBMX+nifedipine. In **(D)**, the pCREB/DAPI-signal ratio was calculated to correct for different cell densities. In both measurements, nifedipine prevented pCREB phosphorylation during differentiation. Scale bars 150 μM.

In the standard differentiation medium, the relative pCREB-fluorescence intensity (ΔF/F_0_) increased from 1.6 ± 0.1 on the first day to 5.4 ± 0.3 on the seventh day (*p* < 0.01). When cultivated under nifedipine, however, pCREB did not significantly increase (1.5 ± 0.2 vs. 1.3 ± 0.2 in control, *p* > 0.1, Figures 7A–C). Because the total cell number differed slightly under the two sets of conditions, the pCREB-fluorescence was normalized to the DAPI-fluorescence intensity, which is proportional to cell number. Thus, the pCREB signal increased from 0.18 ± 0.01 to 1.05 ± 0.04 (*p* < 0.01) without nifedipine, and only from 0.14 ± 0.02 to 0.19 ± 0.03 (*p* > 0.1) with nifedipine (see Figure 7D). Taken together, these results indicate that intracellular Ca^2+^ levels interfere with CREB phosphorylation, which under normal conditions acts synergistically with cAMP to promote neuronal maturation.

To determine whether nifedipine causes significantly high rates of cell death at the used dose, we performed a cell viability assay, as described above (see methods, Cell Viability Assay). To this end, dead and total cells were quantified at the 1st, 3rd, and 5th days *in vitro*. Under IBMX, the yield of dead cells peaked at 2.7 ± 1.3% on the 5th day, which was not significantly different from the 4.5 ± 2.1% observed in the nifedipine cultures on the 1st day (*p* > 0.05). Based on this, we suggest that the difference in cell densities observed across cultures might have been caused by a reduced cell division rate under reduced Ca^2+^. Nevertheless, the possibility that nifedipine might be acting preferentially on neural progenitor cells (NPCs) cannot be completely excluded at this point, as this would also explain why reduced calcium led to lower rates of neurogenesis.

## Discussion

Although much knowledge has been gained in recent years regarding stem cell biology and plasticity, the mechanisms that govern complete neuronal functional maturation remain largely unknown (Scheffler et al., 2005; Ge et al., 2006). In the current study, we present several novel findings pertaining to the differentiation and maturation of SVZ-derived neural progenitor cells (NPCs). First, we found that increasing cAMP levels by inhibiting phosphodiesterase activity is sufficient to support the rapid functional maturation of neuronal progenitors into fully functional neurons; this maturation involves the high-density expression of voltage-gated ion channels, the high-frequency firing of APs and the formation of functional glutamatergic and GABAergic synaptic contacts. Second, we showed that this cAMP-induced acceleration in maturation is dependent on the early up-regulation of L-type Ca^2+^ currents. Finally, early electrical activity and voltage-gated Ca^2+^ influx are critically important for the activation of transcription factors such as pCREB, which induce neuronal outgrowth and synapse formation.

According to (Lipscombe et al., 2004), Ca_v_1 channels, which are expressed in skeletal, cardiac and smooth muscle cells, as well as in neurons and endocrine cells, can mediate numerous cell functions. Ca^2+^ is believed to be involved in controlling cell survival and death (Moulder et al., 2003), as well as in neuronal differentiation (Deisseroth et al., 2003; Lohmann, 2009). Furthermore, intracellular Ca^2+^ signals correlate with the regulation of neuronal gene expression (Toescu and Verkhratsky, 2004), and can activate numerous transcription factors, such as CREB, C/EBTβ, MEF-2, NT-ATc4, NFκ B, and c-fos (Weick et al., 2003). In cortical neurons, Ca_v_1-mediated Ca^2+^ influx stimulates the expression of various genes that regulate neuronal survival and plasticity through CREB phosphorylation (Dolmetsch et al., 2001). Interestingly, when we cultured NPCs without cAMP, MAP2-positive neurons were still generated (6.4 ± 1.2% in basal medium, Figure 1D). However, blocking all Ca^2+^ currents with Cd^2+^ abolished differentiation completely. Our finding confirms that Ca^2+^signaling is critical for proper neuronal differentiation.

In a study of rat dentate gyrus, Stocca et al. (2008) found that young granule cells express a relatively high density of T-type Ca^2+^channels that facilitate the generation of low-threshold Ca^2+^-spikes and boost suprathreshold action potentials with very small excitatory currents. In the current study, we demonstrated the role of L-type calcium channels in neural stem cell differentiation. When we selectively blocked this type of channel with nifedipine, we observed a decrease in the number of mature neurons in culture, whereas applying the L-channel activator Bay K 8644 produced the opposite effect (see also D'Aascenzo et al., 2006). Furthermore, these channels are expressed early in the maturation process. Our data revealed that small Ca^2+^currents were measurable in the differentiation medium after only 1 day; furthermore, blocking them as early as the first day impeded neuronal phenotype acquisition.

Previous work has also highlighted the importance of excitatory stimuli for complete functional differentiation and synaptic maturation (Ge et al., 2006). Newborn granule cells in the dentate gyrus of the adult hippocampus first receive GABAergic synapses before they are innervated by glutamatergic synaptic inputs. GABA initially exerts an excitatory action on newborn neurons thanks to the relatively high chloride ion concentration in the cytoplasm. This GABAergic excitation leads to Ca^2+^-influx, which in turn facilitates neuronal differentiation and dendritic outgrowth in young neurons (Ge et al., 2006). Deisseroth et al. (2004) induced depolarization with glutamate or high K^+^, which resulted in increased neuronal cell production. This effect was then partially reversed when they added nifedipine to the medium, suggesting that the activation of Ca_v_1.2/1.3 (L-type) Ca^2+^ channels plays a role in neurogenesis. Our own data revealed that blocking L-type Ca^2+^ channels with nifedipine had a slightly stronger effect than TTX on the suppression of neuronal differentiation (Figure 4). Taken together, these data confirm the critical role of early electrical excitability and membrane depolarization mediated by Na^+^ channels, Ca^2+^ channels or both. Furthermore, in this study we also showed that these processes may be observed as early as the first few days of neuronal development.

A vast body of work has shown the great neurogenic potential of fetal-derived NSCs and neuronal progenitor cells (Isacson, 2009). In a previous study from our group we demonstrated the superior neurogenic potential of fetal-derived NSC as compared to adult mesenchymal stem cells (Lepski et al., 2010). The role of specific neurotrophic factors and signaling molecules such as BDNF (Leng et al., 2009), NT3 (Yoo et al., 2007), or Wnt (Jagasia et al., 2009) in the functional maturation of neural stem cells has also been reported by several authors. Similarly, many intracellular signaling molecules such as cAMP, Ca^2+^, or GSK3 have been shown to be important for neurogenesis and early neuronal development (Nakagawa et al., 2002; Lohmann, 2009; Hur and Zhou, 2010). Nakagawa et al. (2002) reported that inhibition of phosphodiesterase4 (PDE4) by rolipram strongly increases cognitive functions and the number of newly generated neurons in the hippocampus. In the current study, we showed that inhibition of PDE by IBMX significantly increases the number of MAP2-positive neurons, as well as the expression of voltage-gated Na^+^ and K^+^ channels (and consequently, the firing frequency); these effects are observed within 1 week of treatment. Furthermore, the number of glutamatergic and GABAergic currents was also significantly increased, which may be partially due to the PKA-mediated phosphorylation of synapsins (Kao et al., 2002). Taken together, these results support the notion that cAMP-signaling is critically important for neuronal development (Li et al., 2011). Additionally, the increase in L-type currents is most probably mediated by PKA-mediated phosphorylation (Dai et al., 2009). Our data further indicate that BDNF alone is not sufficient and may act jointly with increased cAMP signaling to effectively support neuronal maturation. In sum, IBMX, cAMP and BDNF appear to constitute the minimal *in vitro* requirements for proper differentiation and maturation of NPCs.

How do cAMP and Ca^2+^-signaling interact to support neuronal differentiation and maturation? It has been reported that embryonic spinal cord neurons generate spontaneous Ca^2+^ and cAMP oscillations (Gorbunova and Spitzer, 2002). Furthermore, blocking Ca^2+^ transients decreases the frequency of cAMP transients and vice versa. In cortical NPCs, we found that increasing cAMP concentration by blocking PDE up-regulated voltage-gated Ca^2+^ currents during the first few days of differentiation. Conversely, blocking voltage-gated Ca^2+^ influx inhibited cAMP-induced neuronal differentiation. Therefore, both cAMP and Ca^2+^ seem to be crucial components that together play a pivotal role in neuronal differentiation and maturation. Our data strongly support a positive feedback model involving sequential increases in cAMP signals and voltage-dependent Ca^2+^ transients during early neuronal differentiation (Willoughby and Cooper, 2007). The Ca^2+^ transients, in turn, lead to CREB activation. As discussed above, the transcription of many proteins important for neuronal growth and synapse formation is regulated during this important stage in neuronal development (Jagasia et al., 2009; Merz et al., 2011).

In conclusion, here we showed that increasing cAMP in the culture system facilitates the generation of mature neurons, while decreasing Ca^2+^influx consistently reduces neuronal production. First, we demonstrated that Ca^2+^ currents in neural stem cells from the fetal subventricular zone are mainly mediated by L-type Ca^2+^channels. Nifedipine did not only block Ca^2+^-influx through L-type channels, but it also effectively inhibited neuronal differentiation. This confirms that voltage-gated Ca^2+^ signaling is critically important for developing NPCs. It seems that Ca^2+^-transients that are elicited during normal cell firing may play a pivotal role in proper neuronal maturation. In neurogenic areas, this appears to be driven by excitatory GABAergic inputs to maturating cells, as has been demonstrated *in vivo* (Tozuka et al., 2005; Ge et al., 2006; Jagasia et al., 2009). Second, we showed that an acute increase in intracellular cAMP leads to an increase in Ca^2+^-influx through L-type channels. Finally, L-type Ca^2+^-blockade impaired CREB phosphorylation, a major signaling hub for neuronal development. Therefore, the data reported herein support a model in which cAMP- and Ca^2+^-dependent pathways positively interact to promote the differentiation of neuronal progenitor cells into fully functional neurons.

To our knowledge, this is the first demonstration of an acute and chronic interaction between Ca^2+^- and cAMP-dependent pathways during *in vitro* neurogenesis. Therefore, these findings represent an important contribution to our understanding of the mechanisms underlying neuronal maturation and to the future development of new restorative strategies based on cell therapy.

### Conflict of interest statement

The authors declare that the research was conducted in the absence of any commercial or financial relationships that could be construed as a potential conflict of interest.
